# An Update on Single-Cell RNA Sequencing in Illuminating Disease Mechanisms of Cutaneous T-Cell Lymphoma

**DOI:** 10.3390/cancers17172921

**Published:** 2025-09-05

**Authors:** Sara Suhl, Alexander Kaminsky, Caroline Chen, Brigit A. Lapolla, Maggie H. Zhou, Joshua Kent, Abigail Marx, Ikenna David Nebo, Geat Ramush, Sophia Luyten, Yoni Sacknovitz, Julie Sung, Christina M. Bear, Celine M. Schreidah, Alejandro Gru, Larisa J. Geskin

**Affiliations:** 1 Vagelos College of Physicians and Surgeons, Columbia University, New York, NY 10032, USA; scs2256@cumc.columbia.edu (S.S.);; 2 Department of Dermatology, Columbia University Irving Medical Center, New York, NY 10032, USA

**Keywords:** cutaneous T-cell lymphoma, CTCL, mycosis fungoides, Sézary syndrome, single-cell RNA sequencing, scRNA-seq, transcriptomic, genomic, tumor microenvironment, biomarkers

## Abstract

Cutaneous T-cell Lymphomas (CTCLs) are a heterogeneous group of non-Hodgkin lymphomas that currently have an incompletely understood pathophysiology and several challenges in both diagnosis and management. Single-cell RNA sequencing (scRNA-seq) is a powerful tool that enables the analysis of gene expression at the individual-cell level, revealing cellular heterogeneity and a complex tumor microenvironment. As single-cell RNA sequencing has become increasingly utilized, we aimed to provide an update on recent notable applications of single-cell RNA sequencing in CTCL and their findings. The included studies highlight the intricate network of interactions in the tumor microenvironment that contributes to tumorigenesis. While CTCL is notoriously heterogeneous, our results identify key markers that prove promising for diagnosis, prognostication, and therapeutic targets.

## Simple Summary

Cutaneous T-cell Lymphomas (CTCLs) refer to a group of rare cancers that affect immune cells in the skin and often the blood. Their exact cause is not entirely understood, and they are difficult to diagnose because they can appear similar to common, non-malignant skin conditions. Recent advances in single-cell RNA sequencing have made it possible for researchers to explore these tumors at an unprecedented level of detail, revealing important differences between CTCL subtypes in how they interact with the immune system. By analyzing these patterns, researchers can begin to explain differences in disease behavior and identify targets for new therapies. This summary highlights how single-cell technologies are transforming our understanding of CTCL and may pave the way for more precise diagnosis and treatment strategies in the future.

## 1. Introduction

Cutaneous T-cell lymphomas (CTCLs) encompass a heterogeneous group of non-Hodgkin’s lymphomas characterized by the clonal proliferation of skin-homing malignant T-cells. Several classification systems have been used to describe CTCL, including the Revised World Health Organization (WHO)–European Organization for Research and Treatment of Cancer (EORTC) classification of primary cutaneous lymphomas (2018) [1], the International Consensus Classification of mature lymphoid neoplasms (2022) [2], and the World Health Organization (WHO) classification of hematolymphoid tumors 5th edition (2022) [3]. The classical subtypes of CTCL are Mycosis Fungoides (MF) and Sézary Syndrome (SS), with the two conditions differing in clinical presentation, tissue involvement, and prognosis. MF is the most common subtype of CTCL, representing approximately 60% of cases. MF is an epidermotropic CTCL that classically exhibits disease progression of skin lesions from patches to plaques to tumors, with potential nodal, blood, or visceral involvement. Histologically, MF is characterized by the presence of small-to-medium-sized T cells with cerebriform nuclei and can also have Pautrier’s abscesses, haloed lymphocytes, and disproportionate epidermotropism. Immunophenotyping reveals loss of mature T cell markers, and T cell receptor (TCR) gene rearrangements are typical. MF subtypes include folliculotropic mycosis fungoides, pagetoid reticulosis, and granulomatous slack skin variants. Overall, MF prognosis is highly variable and largely depends on disease stage, though patients diagnosed at early stages (limited patch/plaque disease) generally have an excellent prognosis and near-normal life expectancy. Sézary Syndrome (SS) is a leukemic form of CTCL that is often associated with erythroderma, lymphadenopathy, and Sézary cells (malignant T cells) present in the skin, blood, and lymph nodes. Unlike MF, SS is associated with a poor prognosis in most patients [1,2,3,4]. While CTCL is uncommon, with an estimated incidence of 8.55 cases per million annually in the US, its incidence has steadily risen over time, most commonly affecting those over age 40 [5].

CTCL pathogenesis is marked by substantial heterogeneity among and within patients. Malignant cells exhibit variable differentiation states, gene expression patterns, and genetic mutations. These variations frequently involve T-cell receptor signaling, cell cycle regulation, and epigenetic remodeling; however, no single unifying genetic or epigenetic alteration has been identified across all cases, and the molecular events leading to malignant transformation, clonal expansion, and disease progression in CTCL remain incompletely understood [6,7,8]. Diagnostic challenges persist due to CTCL’s clinical heterogeneity and resemblance to common benign inflammatory dermatoses [9]. The absence of definitive, clinically applicable biomarkers further complicates early detection and classification. While next-generation sequencing (NGS) approaches have revealed a range of mutational signatures, these findings have yet to translate into reliable diagnostic tools. In recent years, single-cell RNA sequencing (scRNA-seq) has emerged as a powerful approach to address this gap, offering unprecedented insights into specific markers for both diagnosis and therapeutic intervention.

**Figure 1:**
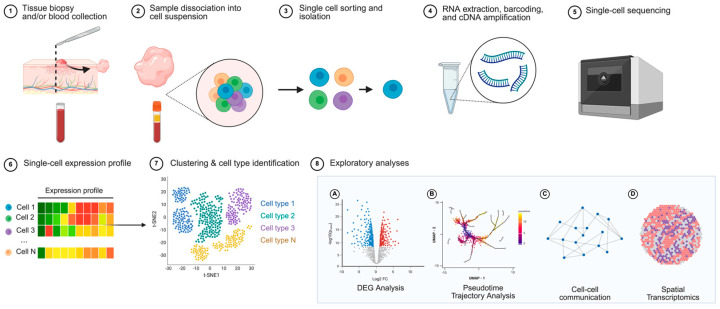
Single-cell RNA sequencing steps. (1) Lesional tissue or blood samples are collected from patients. (2) The collected sample is dissociated into a cell suspension. (3) Cells are sorted and isolated into individual cells. (4) RNA is extracted from individual cells, barcoded, and amplified as cDNA. (5) Cells are sequenced. (6) Each cell’s gene expression profile is quantified, generating a matrix of gene expression values across individual cells. (7) Computational analysis, such as t-SNE (t-distributed stochastic neighbor embedding), is performed to visualize clustering of cells based on similar expression patterns, allowing for identification of distinct cell types (each color in the t-SNE plot represents a different cell type identified through clustering). (8) Exploratory analyses can be performed, including differential gene expression analysis (**A**), trajectory analysis (**B**), cell–cell communication analysis (**C**), and spatial transcriptomic analysis (**D**). Created in BioRender. S, S. (2025) https://BioRender.com/kml8qb1. Accessed 20 July 2025.

Since its first application in 2009, scRNA-seq has grown into an essential tool for understanding disease pathophysiology. A more refined branch of NGS, scRNA-seq allows for high-throughput transcriptomic profiling at the level of individual cells (Figure 1). In contrast to approaches such as bulk RNA sequencing, which captures averaged gene expression across heterogeneous cell populations, scRNA-seq enables the analysis of gene expression from individual cells, granting more granular insights into complex disease processes [10]. In oncological research specifically, scRNA-seq has been particularly impactful in allowing a detailed characterization of tumor heterogeneity and the tumor microenvironment in numerous malignancies, including melanoma [11] and lymphomas [12,13,14]. In doing so, single-cell RNA sequencing studies have collectively yielded novel biomarkers, refined disease classifications, and uncovered new therapeutic targets.

Over the last few years, the adoption of scRNA-seq in CTCL research has transformed the understanding of CTCL by enabling high-resolution dissection of both malignant and non-malignant populations within the CTCL tumor microenvironment. Our group (Gaydosik et al., 2019) utilized single-cell RNA sequencing to demonstrate substantial intra- and inter-tumoral variability in advanced-stage CTCL and identify patient-specific exhaustion-associated transcriptional programs in reactive lymphocytes [15]. Mimitou et al. (2019) applied multimodal expanded CRISPR-compatible cellular indexing of transcriptomes and epitopes by sequencing (ECCITE-seq) to profile peripheral immune cells, utilizing a 49-marker panel of antibodies for in-depth cellular profiling [16]. Rindler et al. (2021) extended these insights by showing that lesion progression was marked by downregulation of CXCR4, CD69, and IL7R specifically within malignant T-cell clones of clinically affected skin, suggesting progressive loss of tissue retention and homeostatic signaling [17]. Complementary work by Herrera et al. (2021) used ECCITE-seq to reveal distinct transcriptional signatures between blood- and skin-derived malignant clones and demonstrated that the skin microenvironment plays an active role in promoting malignant expansion [18]. Additional case-based studies highlight how scRNA-seq can dissect tumor-microenvironment interactions and improve diagnostic clarity. For instance, Jonak et al. (2021) utilized single-cell analysis to distinguish coexisting MF and primary cutaneous follicle center lymphoma clones in a diagnostically ambiguous case [19]. Li et al. (2021) identified novel biomarkers (e.g., CXCL13, VCAM1, CYTOR) and myeloid-fibroblast crosstalk in subcutaneous panniculitis-like T-cell lymphoma (SPTCL) [20], while Borcherding et al. (2019) [21] and Buus et al. (2018) [22] elucidated mechanisms of clonal transition, immunophenotypic plasticity, and drug resistance in Sézary syndrome. Through the use of scRNA-seq in combination with clonality assessment via T cell receptor (TCR) gene rearrangements and immunophenotypic markers, malignant T cells can be reliably identified and studied, enabling precise characterization of their transcriptional profiles, functional states, and interactions within the tumor microenvironment.

These early scRNA-seq studies have laid the foundation for more recent work by establishing the transcriptionally, spatially, and temporally dynamic nature of CTCL. In this review, we will provide an update on findings from notable recent studies utilizing single-cell RNA sequencing in CTCL, with a specific focus on insights into the complex pathophysiology of this incompletely understood disease.

**Figure 2:**
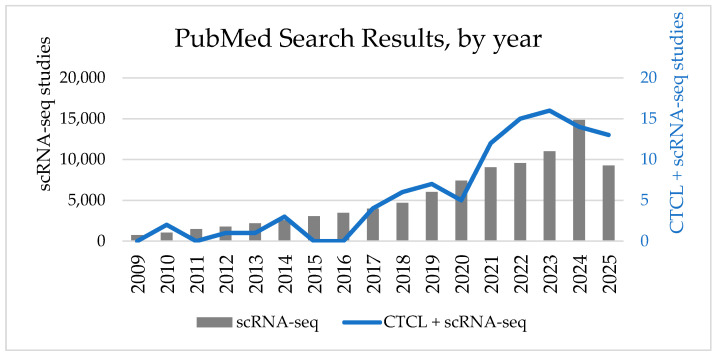
Trends in PubMed Search Results for Single-cell RNA (scRNA-seq) sequencing overall and in Cutaneous T-cell Lymphoma (CTCL) Publications (2009–July 2025). Bar chart (gray) represents the number of PubMed search results for scRNA-seq studies per year (left y-axis, gray), while the line graph (blue) shows the number of search results for combined searches for CTCL and scRNA-seq per year (right y-axis, blue) from 2009 to 2025. (See supplemental methods for exact search terms).

In recent years, the number of published studies on single-cell RNA sequencing has increased dramatically (Figure 2). PubMed search results for single-cell RNA sequencing studies (see Appendix A for exact search terms) have consistently increased each year, with 738 articles published in 2009 and 14,844 published in 2024. Similarly, publications resulting from a search for both CTCL and single-cell RNA sequencing demonstrate similarly increasing adoption, with 99 total studies published since 2009, of which 58 were published since the start of 2022 (see Appendix A for exact search terms used).

Given the remarkable recent surge in CTCL research activity utilizing single-cell RNA sequencing, we focused our review on studies within the last 3 years.

## 2. Methods

**Figure 3:**
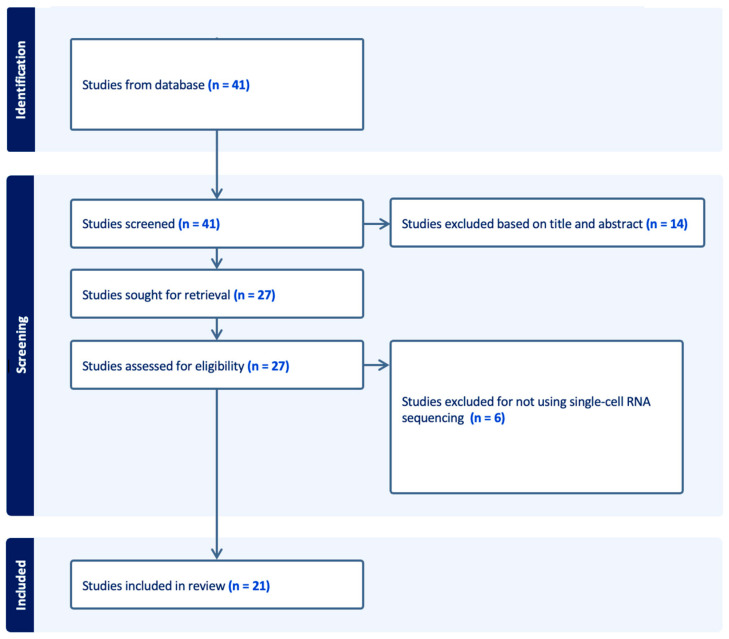
Study Selection Flow Diagram.

We conducted our search in PubMed in July 2025 using a search strategy that included the following terms in the title, abstract, or keywords: (single-cell RNA sequencing) AND (cutaneous T-cell lymphoma) (see Appendix A for full Boolean strings). Results were filtered to include only English-language studies in humans published between 1 July 2022–1 July 2025. Only original investigations using single-cell RNA sequencing to study CTCL were included; thus, we excluded preprints, abstracts or studies without full texts, studies that did not utilize scRNA-seq, and studies of diseases other than CTCL (Figure 3).

We screened titles, abstracts, and full texts using Covidence systematic review software, Veritas Health Innovation, Melbourne, Australia. Available at www.covidence.org. From each included study, we extracted information regarding CTCL subtypes, the number of patients/samples, additional analyses performed, and key findings (see Table A1). Accessed 19 July 2025.

## 3. Findings from Single-Cell RNA Sequencing Studies in CTCL

### 3.1. CTCL Pathogenesis

CTCL’s pathogenesis involves a complex multi-step process driven by genetic, epigenetic, immunologic, and microenvironmental factors. While the evidence does not support a heritable etiology, somatic mutations in some pathways have been implicated, as have environmental factors that induce chronic antigen stimulation. Understanding CTCL’s pathophysiology is made more challenging by its notable inter- and intra-patient heterogeneity. While much remains unknown about CTCL’s pathogenesis, single-cell RNA sequencing has proven to be a useful tool in investigating the intricate pathways involved in lymphomagenesis.

A 2023 study by Harro et al. identified over 200 mutations in hematopoietic stem cells from patients with Sézary syndrome. Peripherally circulating Sézary cells with evidence of recent thymic egress had mutations in key oncogenes, suggesting CTCL originates from mutated lymphocyte progenitor cells that develop T cell receptors in the thymus and then travel to the peripheral circulation to complete their malignant transformation. Additionally, Harro et al. found that the transcriptional profiles of MF and SS cells differentiated into well-defined clusters, supporting the assertion that the two are distinct disease processes [23]. Single-cell sequencing was used by Alkon et al. to clearly differentiate early-stage MF from parapsoriasis, identifying a unique population of NPY(+) innate lymphoid cells in the polyclonal parapsoriasis samples [24]. A 2023 study by Borcherding et al. used scRNA-seq and TCR sequencing to identify distinct quiescent and hyperproliferative populations of Sézary cells, with consistently increased expression of AIRE [25]. Similarly, Ren et al. (2023) found that while diverse, malignant CD4+ T cells typically had mature Th2 differentiation and an exhaustion phenotype. Notably, their analyses identified an “intermediate” degree of mutation and gene expression between benign and malignant CD4+ T cells, consistent with a circulating precancerous population [26]. Jiang et al. similarly found that Sézary cells are distinct from cells of MF lesions, noting 3 distinct subpopulations of Sézary cells (Th1 polarized, intermediate, and Th2 polarized), each with different proliferative potential [27]. Shi et al. used scRNA-seq to identify a distinct cluster of fibroblasts uniquely present in folliculotropic MF, suggesting an underlying mechanism for folliculotropism [28].

Thus, single-cell sequencing has opened the door to new findings related to both the development of and categorization of these malignant entities. Early CTCL is thought to be Th1 skewed in the setting of a reactive infiltrate and epidermotropism, while later stages are characterized by a Th2 phenotype and dermal predominance. The exact mechanism by which this switch occurs is complex and remains incompletely understood, but it appears that the tumor microenvironment (TME) plays a role.

### 3.2. Characterizing the Tumor Microenvironment

Interactions between malignant cells and their environment are of immense importance in understanding cancer biology, as this communication significantly affects disease behavior and therapeutic response. This dynamic is particularly evident in CTCL, as T cells are critical to adaptive immunity and thus frequently interact with other cells in an immune milieu. The skin is a complex, immunologically active organ whose dynamic microenvironment includes lymphocytes, dendritic cells, Langerhans cells, macrophages, keratinocytes, fibroblasts, and commensal microbes [29]. As malignant T cells interact with cells present in both the skin and the blood, each cell influences and is influenced by the evolving tumor microenvironment. Through the release of tumor-derived factors, malignant T cells are able to exploit neighboring cells to facilitate a more favorable environment for continued tumor proliferation. Microenvironmental alterations have been implicated in numerous cellular processes impacting lymphomagenesis, including malignant proliferation and invasion, immunosuppression, and resistance to apoptosis. A 2023 study by Calugareanu et al. reanalyzed publicly available single-cell RNA sequencing data and found that skin in CTCL had a diverse and immunologically active microenvironment with expanded populations of lymphocytes, macrophages, and keratinocytes [30]. In 2023, our group (Gaydosik et al.) conducted a comprehensive transcriptomic analysis of the MF skin tumor microenvironment, revealing the distinct cellular composition, cell–cell interactions, and cellular functioning in MF compared to benign dermatoses or healthy controls. Notable heterogeneity was observed in a host of cellular processes, including immune regulation, metabolism, angiogenesis, and cell trafficking [31]. The tumor microenvironment in CTCL is dynamic and complex, as it promotes the survival and expansion of malignant T cells. A deeper understanding of each cellular component can provide valuable insight into disease pathogenesis and guide future therapeutic interventions.

#### 3.2.1. T Lymphocytes

Recent studies have uncovered distinct characteristics of T lymphocytes across CTCL subtypes, highlighting key markers and shared pathways. Such patterns, including memory phenotypes, cytotoxicity profiles, and apoptotic regulation, may contribute to disease behavior, blood involvement, and progression, providing insight into the pathogenesis of CTCL subtypes.

A 2022 study by Xue et al. analyzed scRNA-seq both from isolated malignant CD4+ cells in PBMCs and from skin samples. PMBC-derived cells had malignant clones expectedly exhibiting a dominant clonotype (TRBV7-2 and TRAAV2), copy number variations, decreased expression of CD7, and aberrantly elevated expression of KIR3DL2 (CD158k) and CD70, which are known to be tumor-associated. These malignant cells had evidence of active proliferation, with significant upregulation of genes associated with T cell activation (TNFRSF4), growth (PIM2, PRDX, NPM1), the cell cycle (CCND2, CCND3, and DUSP4), and cell survival (BIRC3). These central memory phenotype cells (SELL+-CCR7+CD27+TCF7+S100A) were only weakly positive for CCR10 and CCR4 (skin homing). In the skin cell analysis, of all the diverse cells identified in the skin microenvironment (including lymphatic endothelial cells, macrophages, keratinocytes, fibroblasts, melanocytes, hair follicle cells, vascular endothelial cells, vascular smooth muscle cells), T cells were the most heterogeneous between samples. Malignant CD4+ cells exhibited upregulated CCR7, CD27, and SELL, but also expressed CCR4 and CD69 (skin-homing molecules), and NR4A1 (tissue-resident associated gene), thus sharing characteristics of both central memory phenotype and tissue-resident phenotype. Overall, the study found that skin-derived Sézary cells had more mature phenotypes than blood-derived cells, but specific markers were shared by both (TOX, DNM3, KLHL42, PGM2L1, and SESN3) [32]. These findings may also provide evidence of T cell phenotypic plasticity, a phenomenon with significant implications for immune-mediated diseases, including CTCL [33].

A 2025 study by Chennareddy et al. [34] analyzed single-cell RNA sequencing of classic CD4+ advanced-stage mycosis fungoides, TCR-γ/δ mycosis fungoides, and primary cutaneous CD8 aggressive epidermotropic cytotoxic T-cell lymphoma (pcAECyTCL). They found that malignant clones from TCR-γ/δ+ MF and pcAECyTCL had increased expression of NKG7, CTSW, GZMA, and GZMM (cytotoxic markers) as well as CD69, CXCR4, NR4A1 (associated with a tissue resident phenotype), and were not associated with blood involvement. Upregulation of DDIT4 (associated with cellular stress, proliferation, and negative regulation of apoptosis) was seen in both TCR-γ/δ and pcAECyTCL samples. pcAECyTCL clones were associated with upregulation of CCL5 (chemokine associated with Type 1 pathway), ERN1 (promotes CD8+ secretory and effector functioning), tumorigenesis promoter ARL4C (Wnt/β-catenin and epidermal growth factor/Ras target), TNF-α (pro-inflammatory), and GZMM, NCR3, and PRF1 (associated with cytotoxicity). pcAECyTCL was also associated with lower PTPRC expression, which may lead to uninhibited Janus kinase 2 signaling. TCR-γ/δ clones were associated with upregulated GNLY (granulysin), KLRC1 (NK receptor), TMIGD2 (associated with tissue-resident memory T cells), and LGALS3 (associated with skin tissue residency). Contrastingly, advanced stage CD4+ MF clones uniquely expressed SELL, CCR7, LEF1 (central memory T-cell markers), and had upregulation of genes implicated in T cell migration and chemotaxis (LAIR2, TIAM1, RIPOR2). As the CD4+ MF subtype was associated with blood involvement, its uniquely upregulated pathways suggest a possible mechanism behind the ability of malignant CD4+ clones to travel between tissue types. Malignant clones of the CD4+ MF subtype also uniquely expressed CTCL markers (GIMAP7, GIMAP4, HACD1, IGFL2, KLHL42, PGM2L1, SESN3) and had upregulated PASK (serine/threonine kinase associated with naïve and central memory T cells) and TMEM243 (transmembrane protein associated with multi-drug resistance in malignancies) [34]. Another 2025 study comparing erythrodermic CTCL to chronic idiopathic erythroderma, atopic dermatitis, psoriasis, and healthy controls found that erythrodermic CTCL had expanded CD4+ malignant cells with a CCR7+SELL+ central memory phenotype [35].

A 2025 study by Jung et al. [36] utilized single-cell transcriptomics to distinguish intraepidermal T cells in early-stage mycosis fungoides (MF) from benign skin conditions, such as psoriasis and chronic spongiotic dermatitis. The researchers identified 41 differentially expressed genes, predominantly involved in Th17 differentiation, T-cell receptor signaling, and apoptosis. Granulysin (GNLY) and FYN binding protein 1 (FYB1) showed the highest fold changes and emerged as potential biomarkers to differentiate early MF from benign mimickers, demonstrating notable diagnostic performance (GNLY: sensitivity 67.9%, specificity 93.6%, AUC 0.86; FYB1: sensitivity 73.2%, specificity 69.2%, AUC 0.79) [36]. A 2024 study by Luo et al. analyzed publicly available scRNA-seq datasets of MF, SS, and healthy controls, finding increased expression of CDK9 (which promotes retinoic acid receptor α degradation via HUWE1 (E3 Ligase) recruitment) in malignant T-cell clusters of both skin and blood [37]. A 2024 study by Srinivas showed that malignant cells overexpressed galectins (LGALS1, LGALS3), S100 genes (S100A4, S100A6), and keratins (KRT81, KRT86) [38].

**Figure 4:**
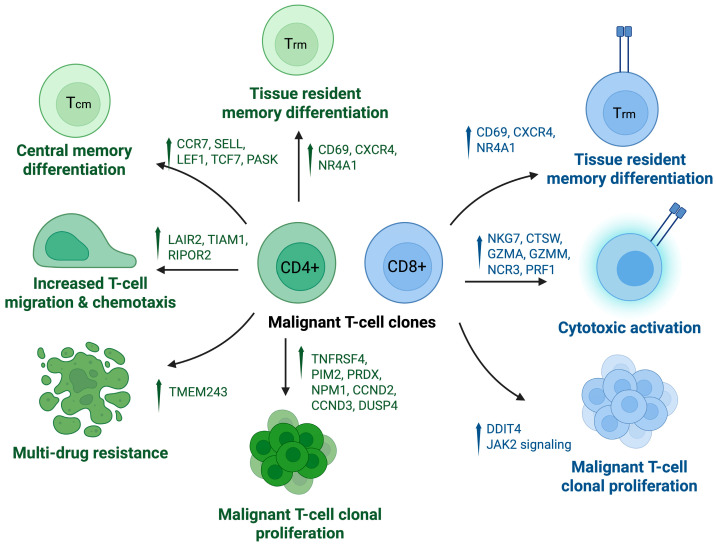
Signaling pathways of CD4+ and CD8+ malignant T-cell clones. Schematic illustrating functional and transcriptional pathways enriched in malignant CD4+ and CD8+ T-cell clones. Malignant CD4+ clones (left, green) exhibit upregulation of genes associated with central memory (Tcm) differentiation (e.g., CCR7, SELL, LEF1, TCF7, PASK), tissue-resident memory (Trm) differentiation (CD69, CXCR4, NR4A1), increased migration and chemotaxis (LAIR2, TIAM1, RIPOR2), multi-drug resistance (TMEM243), and clonal proliferation (TNFRSF4, PIM2, PRDX, NPM1, CCND2, CCND3, DUSP4) are also upregulated. Malignant CD8+ clones (right, blue) similarly show markers of Trm differentiation (CD69, CXCR4, NR4A1), activation of cytotoxic effectors (NKG7, CTSW, GZMA, GZMM, NCR3, PRF1), and clonal proliferation (DDIT4, JAK2 signaling). Created in BioRender. S, S. (2025) https://BioRender.com/ezs4hew. Accessed 20 July 2025.

These studies highlight the important and dynamic role of T lymphocytes in CTCL pathogenesis, offering insight into the differential patterns among CTCL subtypes. More aggressive variants are often associated with cytotoxic and tissue-resident features, while CD4+ MF are more associated with central memory traits that are seen with blood involvement and migration. Synthesizing these patterns allows for greater understanding of specific disease behavior, guiding prognosis and treatment strategies (Figure 4).

#### 3.2.2. B Lymphocytes

**Figure 5:**
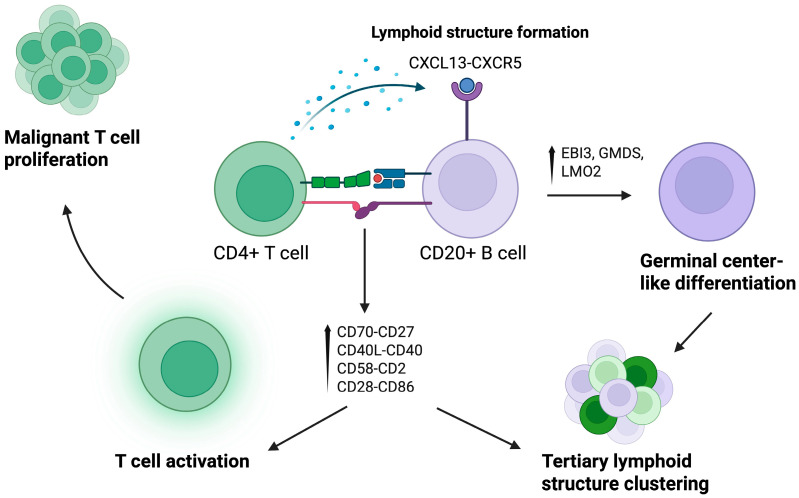
Cell–cell Interaction and Signaling Pathway of CD4+ T cells and B cells in the Tumor Microenvironment. Direct T and B cell interactions via co-stimulatory receptor-ligand pairs (CD70–CD27, CD40L–CD40, CD58–CD2, CD28–CD86) lead to T cell activation and support malignant T-cell proliferation. Concurrently, CXCL13–CXCR5 signaling promotes lymphoid structure formation. Upregulation of EBI3, GMDS, and LMO2 in B cells suggests germinal center-like differentiation. These coordinated events culminate in the clustering of tertiary lymphoid structures, which may further sustain malignant proliferation and immune modulation within the tumor niche. Created in BioRender. S, S. (2025) https://BioRender.com/6fky8v4. Accessed 20 July 2025.

B lymphocytes are emerging as contributors to CTCL pathogenesis, interacting with malignant T cells and shaping the tumor microenvironment more meaningfully than previously appreciated. In 2024, Li et al. [39] published a pivotal study of their work generating the largest scRNA-seq skin cell atlas of CTCL, with 45 patients. To do so, they performed single-cell RNA sequencing, T cell receptor (TCR) sequencing, and integrated publicly available single-cell RNA sequencing data, with scRNA-seq and bulk RNA-seq data of atopic dermatitis, psoriasis, and healthy controls used as comparisons. Their findings revealed that B cells were significantly enriched in the lesional skin and closely interacted with malignant T-cells. Malignant T cells were found to highly express CXCL13, a gene encoding a B lymphocyte chemoattractant. Notably, increased CXCL13 expression was not seen in the healthy control, atopic dermatitis, or psoriasis controls. Spatial transcriptomics with immunofluorescence confirmed physical proximity and direct contact between CD20^+^ B cells and CD4+ malignant T cells, with multiple costimulatory ligand-receptor interactions, including those important in B cell recruitment (CD70-CD27, CD40LG-CD40) and the formation of lymphoid structures (CXCL13–CXCR5). B-cells of multiple subtypes (naïve, memory) were found to aggregate together in a formation reminiscent of tertiary lymphoid structures. Germinal center-like B-cells expressed EBI3, GMDS, and LMO2. Overall, the study found that B cells may play a prominent role in promoting tumor growth in CTCL, and determined that B cell enrichment of the tumor microenvironment could even be associated with poorer prognosis [39] (Figure 5).

#### 3.2.3. Keratinocytes

**Figure 6:**
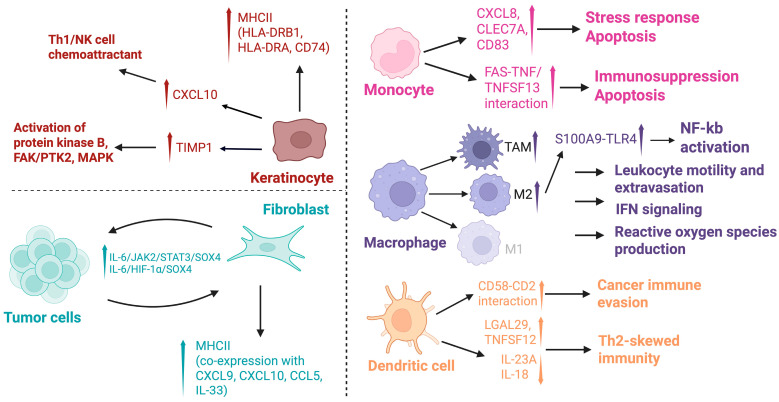
Keratinocyte, Fibroblast, Monocyte, Macrophage, and Dendritic Cell Interactions in the Tumor Microenvironment. Keratinocytes (top left panel) upregulate MHCII, attract Th1/NK cells, and activate protein kinase B, FAK/PTK2, and MAPK pathways. Fibroblasts (bottom left panel) communicate with tumor cells via IL-6/JAK2/STAT3 and IL-6/HIF-1α pathways, inducing MHCII and chemokine expression. Among the myeloid cells (right panel), monocytes express CXCL8 and CLEC7A and engage in FAS–TNF signaling, contributing to stress response, immunosuppression, and apoptosis. M2 Macrophages and Tumor Associated Macrophages (TAMs), activating NF-kB and promoting increased leukocyte motility, IFN signaling, and reactive oxygen species production. Dendritic cells mediate cancer immune evasion via CD58-CD2 interactions and promote Th2-skewed immunity with upregulated LGAL29, TNFSF12, and downregulated IL-23A and IL-18. Created in BioRender. S, S. (2025) https://BioRender.com/prjxamo. Accessed 20 July 2025.

Keratinocytes have increasingly been associated with CTCL pathogenesis and may offer insight into differentiating CTCL from benign or reactive processes. The 2023 study by Calugareanu found that skin in CTCL was associated with an increased proportion of keratinocytes, relative to benign dermatoses or healthy controls [30]. Our prior work identified 20 genes expressed almost exclusively in keratinocytes from MF samples, with immunofluorescent microscopy validating the presence of KRT6A^+^S100A8^+^ in the epidermis of advanced MF but not healthy controls [31]. A 2025 study by Cabrera-Perez et al. found that keratinocyte-specific alterations of IL-4R and IL-13RA1 could be seen in both CTCL and atopic dermatitis, suggesting dupilumab may unmask or spur CTCL progression by blocking IL-13 receptor, thus increasing IL-13 in the local environment [40]. In Chennareddy et al.’s 2025 study, both chronic idiopathic erythroderma and erythrodermic CTCL had keratinocytes and fibroblasts with upregulated MHC II genes (HLA-DRB1, HLA-DRA, and CD74), likely in response to IFN-γ [35]. Chennareddy et al. also found that keratinocyte activation markers were upregulated in CD4+ MF and TCR-γδ^+^ MF, but not pcAECyTCL, suggesting a potential etiology for pcAECyTCL’s classic ulceronecrotic appearance [34]. Calugareanu found that keratinocytes had increased expression of CXCL10 (chemokine that attracts Th1 and NK cells) and TIMP-1 (leads to activation of protein kinase B, FAK/PTK2, and MAPKs) [30]. Together, these findings highlight the role of keratinocytes in CTCL pathogenesis, as they can influence chemokines and inflammatory signals, possibly helping distinguish CTCL from other benign skin conditions. Such understanding can provide diagnostic and therapeutic guidance across CTCL subtypes (Figure 6).

#### 3.2.4. Fibroblasts

Recent studies have also explored the role of fibroblasts in CTCL, particularly in advanced stages, where fibroblasts may be implicated in tumor progression and metastasis. A 2024 study by Zhao et al. found that hyperactivity of inflammatory cancer-associated fibroblasts was a marker of advanced-stage MF, with a proposed mechanism of bi-directional interaction in which tumor cells cause inflammatory cancer-associated fibroblast proliferation, thus improving malignant T-cells’ ability to metastasize via the IL-6/JAK2/STAT3/SOX4 or IL-6/HIF-1α/SOX4 pathways [41]. Calugareanu et al. noted upregulation of A8 and S100A9 (calcium-binding proteins) in fibroblasts as well as endothelial cells and macrophages [30]. Shi et al. identified a cluster of fibroblasts uniquely found in folliculotropic MF, with markers (ADAMTS8, ADH1B, CCL19, CCL26, CP, CRABP1, PLA2G2A, PTX3, RBP5, STEAP2) associated with cell adhesion, chemotaxis, and ECM structure [28]. These studies underscore the active role of fibroblasts in malignant T cell metastasis and disease progression, providing more insight into future therapeutic targets and strategies (Figure 6).

#### 3.2.5. Myeloid Cells

Our group (Gaydosik et al., 2023) found that myeloid cells were present at an increased frequency in MF samples, compared to healthy controls. Phenotypic changes found in advanced-stage MF included plasmacytoid dendritic cells (pDC) populations, M2-like macrophages, and TAM expansion [31].

Monocytes are also an important component of the tumor microenvironment, contributing to signal transduction, apoptosis, and lymphomagenesis. A 2024 study by Jiang et al. found that monocytes from SS patients had heightened expression of CXCL8, CLEC7A, and CD83 and are involved in pathways relating to chemical stress response, negative regulation of signal transduction, and apoptosis. Monocytes that interacted with Sézary cells uniquely had binding between FAS with TNF and TNFSF13, leading to a shift toward proapoptotic immunosuppression. There was also increased expansion of intermediate monocytes that are less responsive to cytokine signaling. CD4+ T-cells emerged alongside the accumulation of dysfunctional monocytes with impaired fragment crystallizable γ-dependent phagocytosis, supporting an important role of monocytes in lymphomagenesis through their reduced ability to eradicate Sézary cells [42]. These findings highlight the role of monocytes in the CTCL microenvironment, as they can support malignant T-cell survival and promote disease progression. Their influence on immunosuppression and lymphomagenesis offers potential avenues for future therapeutic targets.

As antigen-presenting cells, dendritic cells interact closely with T cells to trigger immune responses or maintain immune homeostasis. Du et al. noted that LAMP+ conventional dendritic cells (cDC) were important in mediating immunosuppression by interacting with malignant CD8+ T cells [43]. LAMP3+ cDC cells were enriched in CTCL, with notable interactions of TGFB1-TGFβ, NOTCH1-TNF, CD47-SIRPA between cDCs and malignant T cells [43]. Our group’s work found that MF-specific pDC had upregulation of pathways associated with LRX/RXR activation, IFN and lymphotoxin β signaling, NRF2-mediated oxidative stress, and HIF-1α and ferroptosis signaling [31].

Macrophages are also key players in the immune milieu and can adopt pro-inflammatory (M1-like) or immunosuppressive (M2-like) phenotypes, influencing tumor progression, immune evasion, and therapeutic response. Du et al. found that M2 macrophages contributed to tumor growth through S100A9 upregulation and NF-kb activation [43]. Our group found prominent expansion of M2 macrophages and tumor-associated macrophages (TAM), and noted that a macrophage cluster specific to MF had upregulated processes related to leukocyte motility and extravasation, reactive oxygen species production, cachexia, and IFN signaling [31] (Figure 6).

### 3.3. Drug Interactions

Characterizing interactions between malignant T cells and therapeutic pathways provides critical insight into the underlying mechanisms of disease progression, immune evasion, and therapeutic resistance. These interactions not only reflect the biological behavior of malignant clones within the tumor microenvironment but also help elucidate how treatments modulate, or fail to modulate, the immune landscape. Du et al. (2022) identified that upregulated S100A9 increased CTCL tumor growth via the NF-kB pathway, and then demonstrated that tasquinimod inhibited CTCL tumor growth in vitro by blocking S100A9 and TLR4 [43]. A 2023 study by Borcherding et al. compared malignant T cell populations in an individual patient during their treatment with photopheresis and a histone deacetylase inhibitor, finding that FOXP3 expression increased as treatment progressed. Given FOXP3’s role in regulating regulatory T cells, the study highlights a possible mechanism of immune evasion [25]. Ren et al. highlighted CD82 and JAK as potential therapeutic targets, given their role in the survival and proliferation of malignant T cells [26]. Gao et al. (2023) reported a case of a patient with disease hyperprogression on a PD-1 inhibitor, with single-cell RNA sequencing revealing proliferating CD4+ malignant T cells with an exhausted phenotype and a somatic mutation in PRKCQ (leading to constant activation of T cell activation/NF-κB pathway) [44]. However, this observation may be more useful as a mechanistic insight rather than evidence of a class-wide phenomenon, given the lack of broad clinical validation and CTCL’s diversity of molecular alterations. Notably, this case suggested that PD-1 may serve as a tumor suppressor of malignant T cells with TCR activation [44]. Such insights support future trial designs incorporating biomarker-guided patient/treatment selection and ongoing sample collection from patients while on treatment. While the role of immune checkpoint inhibitors (ICI) in treating CTCL remains incompletely characterized, recent studies have found ICI, including PD-1, to be promising [45]. Recent clinical trials have studied Nivolumab [46,47], Pembrolizumab [48,49], Durvalumab [50], and TTI-621 (targeting CD47) [51] either alone or as combination therapies.

Costanza et al. (2025) investigated CD74 (major histocompatibility complex class II chaperone) as a potential therapeutic target using multiple methods, including scRNA-seq [52]. They found that CD74 was expressed in both MF and SS, and chemotherapeutic antibody-drug conjugates targeted to CD74 were effective in killing CTCL cells in vitro and in an animal model [52]. A 2022 study by Su et al. noted an upregulation of KLHL42 in skin and blood-derived Sézary cells. Using luciferase reporter, gene knockdown, and functional assays, they determined that GATA3 transcriptionally activates KLHL42 in SS, and its silencing promotes apoptosis and inhibits rapid CTCL proliferation, making KLHL42 a promising but currently unutilized therapeutic target [53]. In the context of CTCL, a disease with notable phenotypic heterogeneity and variable treatment responses, dissecting therapeutic interactions can uncover actionable targets, inform personalized therapeutic strategies, and guide the development of novel immunomodulatory agents. Ultimately, such studies are essential for translating molecular findings into clinically meaningful interventions that improve patient outcomes.

### 3.4. Additional Works

While our formal review did not include conference abstracts, it is worth noting that many useful insights regarding scRNA-seq in CTCL have been presented at national and international conferences in recent years. These presentations have discussed findings including: identifying progressive T-cell expansion across disease stages from folliculotropic MF to large cell transformation (LCT) [54], proposing exportin-1 (XPO1) as a potential target by highlighting therapeutic dependency in a single-cell atlas [55], exploring the gene expression patterns and tumor microenvironment interactions of malignant T cells [56,57,58], and exploring race and ethnicity related transcriptional differences in early MF [59].

### 3.5. Challenges and Future Directions

Despite its promise, scRNA-seq is not without its limitations. While ongoing technological advances and the decreasing cost of genomic sequencing continue to generate excitement around scRNA-seq’s potential, several key challenges remain. First, the degree of heterogeneity inherent in malignant CTCL populations means that single-cell sequencing alone is currently insufficient for conclusively diagnosing the disease [15,21,23]. Despite the significant advances in understanding CTCL’s pathogenesis, as demonstrated in the reviewed articles, a complete picture of CTCL’s mechanistic underpinnings remains elusive.

Although the price of genomic sequencing is much lower and the time required to run analyses is much shorter than when the technology was still in its infancy, it is still not practical, from a clinical perspective, to utilize this technology for all patients with clinical suspicion for CTCL. Thus, its clinical utility, at this stage, is quite limited in scope. Due to the resource-intensive nature of single-cell RNA sequencing, studies often have limited sample sizes, limiting the generalizability of findings [60,61]. scRNA-seq is also less sensitive to genes with low expression levels, making targeted CTCL-specific gene panels a useful tool used by several studies in our review. While these methods enhance feasibility, they still require further validation and optimization.

Recently, more studies have begun utilizing publicly available scRNA-seq data, a trend that will likely continue as more data becomes available. While re-analysis of public data allows researchers to more efficiently analyze larger numbers of patient samples, it is not without its challenges. The heterogeneity of sample processing and RNA-seq experimental protocols introduces artifacts and confounding variables that prove problematic for grouped analyses [30,62]. Additional developments in the field include multiomic analyses, such as ECCITE-seq and spatial transcriptomics [16,63]. These technologies, in combination with scRNA-seq, allow for an even more comprehensive and granular characterization of the tumor microenvironment and transcriptomic underpinnings of disease. While scRNA-seq has been pivotal in deepening our understanding of CTCL’s pathophysiology and identifying molecular targets, these advanced technologies prove promising in the study of CTCL.

Emerging computational technologies, such as trajectory inference methods, including pseudotime analysis implemented in tools like Monocle, offer a view into the progression through intermediate states of malignancy, which may represent key therapeutic targets. In parallel, tools including CellPhoneDB enable systemic mapping of ligand-receptor interactions between malignant T cells and their local microenvironment, providing context on how intercellular interactions may drive disease progression and immune evasion. Together, these tools expand the potential of scRNA-seq beyond static snapshots, offering more context into malignant progression [64]. Artificial intelligence and machine learning approaches also represent an exciting frontier. These tools are increasingly used to analyze high-dimensional scRNA-seq data, improving dimensionality reduction, clustering, and prediction of cell states [65]. In CTCL and other cancers, these models have potential uses in molecular subtyping, biomarker discovery, and even predicting therapeutic responses [66].

Looking ahead, as these advanced sequencing technologies become more affordable and accessible, their increased use promises rapid progress in our understanding, diagnosis, and treatment of complex diseases, such as CTCL. Combining scRNA-seq findings with long-term clinical data and applying these insights to therapy planning will be crucial in the next phase of translational research for CTCL.

## 4. Conclusions

There is much work that remains to be done in the study of CTCL, a disease that clinically mimics many other conditions, is often characterized by non-diagnostic biopsies, has incompletely understood pathogenesis, and can be refractory to treatment and cause significant morbidity. Single-cell sequencing, a rapidly evolving technology that has allowed for important advances in the understanding of many disease entities, has attracted interest as a potential tool to advance our understanding of this condition.

This review highlights the increasing adoption of scRNA-seq in unraveling CTCL’s complex pathophysiology and aiding in diagnostic, prognostic, and therapeutic markers. Its complex tumor microenvironment and significant interpatient and intratumor heterogeneity make CTCL a disease that is well-suited to the benefits of single-cell RNA sequencing (scRNA-seq) technology. Due to its complex tumor microenvironment and considerable variability both between and within tumors, CTCL greatly benefits from single-cell RNA sequencing (scRNA-seq) technology. Through detailed profiling, researchers have identified new malignant subclones, uncovered immunological and microenvironmental heterogeneity, and suggested potential biomarkers and therapeutic targets.

Decoding CTCL’s complex oncogenic and immunologic pathophysiology benefits not only patients, as these discoveries may lead to prevention, early detection, or treatment, but also serves as a model that can be used to better understand immunology and oncogenesis [29]. Nonetheless, scRNA-seq is not yet suitable for routine clinical use. Obstacles such as high cost, computational requirements, limited sensitivity for rare transcripts, and lack of standardized pipelines still remain [60,61]. However, with ongoing progress in multi-modal technologies and AI-based analytics, these challenges may soon be overcome. Ultimately, scRNA-seq has not only improved our understanding of CTCL biology but also shows the power of single-cell technologies in uncovering the mechanisms of cancer and immune dysregulation. As we refine these tools and incorporate them into translational workflows, they have the potential to enhance diagnosis, monitoring, and treatment strategies for patients with CTCL and beyond.

## Data Availability

Data available upon request of study authors.

## Abbreviations

The following abbreviations are used in this manuscript:

AUC	Area Under the Curve
AI	Artificial Intelligence
AD	Atopic Dermatitis
CD	Cluster of Differentiation (e.g., CD4, CD8, CD20)
cDC	Conventional Dendritic Cell
pDC	Plasmacytoid Dendritic Cell
CTCL	Cutaneous T-cell Lymphoma
ECCITE-seq	Expanded CRISPR-compatible Cellular Indexing of Transcriptomes and Epitopes by sequencing
FAK/PTK2	Focal Adhesion Kinase/Protein Tyrosine Kinase 2
HUWE1	HECT, UBA and WWE domain containing E3 ubiquitin protein ligase 1
HIF-1α	Hypoxia-inducible factor 1-alpha
IFN-γ	Interferon gamma
JAK2	Janus Kinase 2
KLHL42	Kelch-like family member 42
KCs	Keratinocytes
MHC II	Major histocompatibility complex class II
MAPKs	Mitogen-Activated Protein Kinases
MF	Mycosis Fungoides
NGS	Next-generation sequencing
NF-κB	Nuclear Factor kappa-light-chain-enhancer of activated B cells
(pcAECyTCL)	Primary Cutaneous CD8+ Aggressive Epidermotropic Cytotoxic T-cell Lymphoma
PBMCs	Peripheral blood mononuclear cells
PRKCQ	Protein Kinase C theta (gene)
PKCθ	Protein Kinase C theta (protein)
RARα	Retinoic Acid Receptor alpha
SS	Sézary Syndrome
scRNA-seq	Single-cell RNA sequencing
SPTCL	Subcutaneous Panniculitis-like T-cell Lymphoma
Th1/Th2/Th17	T helper cell types 1, 2, and 17
TCR	T-cell receptor
TME	Tumor microenvironment
TLS	Tertiary Lymphoid Structures
TNF-α	Tumor Necrosis Factor alpha

## Appendix A. Search Strategy

The PubMed search terms used to identify single-cell RNA sequencing studies were as follows: (“Single-Cell Analysis”[Mesh] OR “Single-Cell Gene Expression Analysis”[Mesh] OR “single cell analysis”[Title/Abstract] OR “single-cell gene expression analysis”[Title/Abstract] OR “single cell gene expression analysis”[Title/Abstract] OR “single-cell RNA sequencing”[Title/Abstract] OR “single cell RNA sequencing”[Title/Abstract] OR “single-cell RNA seq”[Title/Abstract] OR “single cell RNA seq”[Title/Abstract] OR “single-cell RNA”[Title/Abstract] OR “single cell RNA”[Title/Abstract] OR scRNA*[Title/Abstract] OR “sc-RNA”[Title/Abstract] OR “sc-RNA-sequencing”[Title/Abstract] OR “sc-RNA-seq”[Title/Abstract] OR “scRNA-seq”[Title/Abstract] OR “scRNA-sequencing”[Title/Abstract] OR “Sc-rna-sequencing”[Title/Abstract] OR “sc-rna-seq”[Title/Abstract] OR transcriptom*[Title/Abstract]).

The PubMed search terms used to identify single-cell RNA sequencing studies in CTCL specifically: (“cutaneous t cell lymphoma”[Title/Abstract] OR “cutaneous t cell lymphoma”[Title/Abstract] OR (“cutaneous”[Title/Abstract] AND (“T”[Title/Abstract] OR “T-cell”[Title/Abstract]) AND (“lymphoma”[Title/Abstract] OR “lymphoproliferative”[Title/Abstract])) OR “mycosis fungoides”[Title/Abstract] OR “Pagetoid Reticulosis”[Title/Abstract] OR “Woringer-Kolopp”[Title/Abstract] OR “Ketron-Goodman”[Title/Abstract] OR “Granulomatous slack skin”[Title/Abstract] OR “Sezary syndrome”[Title/Abstract] OR “Primary cutaneous CD30-positive lymphoproliferative”[Title/Abstract] OR “primary cutaneous cd30 lymphoproliferative”[Title/Abstract] OR “Lymphomatoid papulosis”[Title/Abstract] OR “Primary cutaneous anaplastic large cell lymphoma”[Title/Abstract] OR “Subcutaneous panniculitis-like T-cell lymphoma”[Title/Abstract] OR “Lymphoma, T-Cell, Cutaneous”[Mesh] OR “Mycosis Fungoides”[Mesh] OR “Subcutaneous panniculitis-like T-cell lymphoma” [Supplementary Concept]) AND (“Single-Cell Analysis”[Mesh] OR “Single-Cell Gene Expression Analysis”[Mesh] OR “single cell analysis”[Title/Abstract] OR “single-cell gene expression analysis”[Title/Abstract] OR “single cell gene expression analysis”[Title/Abstract] OR “single-cell RNA sequencing”[Title/Abstract] OR “single cell RNA sequencing”[Title/Abstract] OR “single-cell RNA seq”[Title/Abstract] OR “single cell RNA seq”[Title/Abstract] OR “single-cell RNA”[Title/Abstract] OR “single cell RNA”[Title/Abstract] OR scRNA*[Title/Abstract] OR “sc-RNA”[Title/Abstract] OR “sc-RNA-sequencing”[Title/Abstract] OR “sc-RNA-seq”[Title/Abstract] OR “scRNA-seq”[Title/Abstract] OR “scRNA-sequencing”[Title/Abstract] OR “Sc-rna-sequencing”[Title/Abstract] OR “sc-rna-seq”[Title/Abstract] OR transcriptom*[Title/Abstract]).

**Table A1:** Included studies.

Study	CTCL Types	# CTCL Patients in Single-Cell Analysis	Key Analysis Types	Key Findings
Cabrera-Perez et al., 2025 [40]	Advanced CTCL	5	scRNA-seq Bulk RNA-seq	- Changes in IL-4RA and IL-13RA1 expression in CTCL and AD epidermal layers, most divergent in keratinocytes
Costanza et al., 2025 [52]	SS, MF, LyP, pcALCL	10	scRNA-seq TCR-seq, PCR, IHC, IF, FC, CODEX, DNA methylation profiling	- CD74 is highly expressed in CTCL across subtypes, correlating with CD74 DNA hypomethylation in CTCL cell lines. - Targeting CD74 was effective in killing CTCL cell-lines in vitro and in vivo
Chennareddy et al., 2025 [35]	Erythrodermic MF, SS	8	scRNA-seq, TCR-seq, IF	- eCTCL associated with marked expansion of malignant CD4+ T-cell clones with a CCR7^+^ SELL^+^ central memory phenotype. - Both eCTCL and CIE display upregulation of MHC class II–related genes (HLA-DRB1, HLA-DRA, CD74) in keratinocytes and fibroblasts
Jung et al., 2025 [36]	MF (CD4+, patch stage)	8	scRNA-Seq, CosMx spatial molecular imager, IHC, protein–protein interaction, GSEA	- 41 upregulated differentially expressed genes (DEGs) in MF - Apoptosis, Th17 cell differentiation, and TCR signaling pathways were enriched in MF. - GNLY and FYB1, DEGs with the highest fold-change values, were selected as potential diagnostic biomarkers for MF.
Alkon et al., 2025 [24]	MF (Advanced & early stage)	7	scRNA-seq, TCR-seq	- Clonal LPD lesions (esMF) exhibit CD7/CD27 downregulation and IL26 upregulation. - Polyclonal PP lesions have unique NPY+ innate lymphoid cells.
Chennareddy et al., 2025 [34]	CD4+ MF, TCR-γδ+ MF, pcAECyTCL	14	scRNAseq, TCR-seq, trajectory analysis	- CD4+ MF: Central memory T-cell phenotype (SELL, CCR7, LEF1), blood involvement, upregulated CCR4/KIR3DL2 (therapeutic targets). - TCR-γδ+ MF & pcAECyTCL lymphoma: Tissue-resident phenotype (CD69, CXCR4, NR4A1), cytotoxic markers (NKG7, GZMA, GZMM), no blood involvement. - pcAECyTCL: Lack of keratinocyte activation (S100A, KRT16), type 1 immune skewing, ulceronecrotic lesions.
Zhao et al., 2024 [41]	Advanced MF	2	scRNA-seq, CNV, trajectory analysis, SCENIC analysis, cell–cell communication, IHC, IF	- Malignant cells transitioned from a TRM-dominant to TCM-dominant state during progression from patch/plaque to tumor stage. - SOX4 and IL-6 highly expressed in tumor stage lesion. - LR interactions between T cells and fibroblasts included MDK–LRP1, PDGFD–PDGFRB, CXCL12–CXCR4, TGFBR1–TGFBR3, IGF1–IGF1R, FGF7–FGFR1, CXCL12–DPP4, and MIF–EGFR interactions. - Increased TWIST1 and MMP2 expression in tumor-stage MF
Luo et al., 2024 [37]	MF, SS	20	scRNA-seq, Bulk RNA-seq, proteomic screening	- Elevated CDK9 malignant T cell cluster with a unique actively proliferating feature. - Inhibition, depletion or degradation of CDK9 significantly reduces CTCL cell growth in vitro and in murine models. - CDK9 also promotes degradation of retinoic acid receptor α (RARα) via recruiting the E3 ligase HUWE1. Co-administration of CDK9-PROTAC (GT-02897) with all-trans retinoic acid(ATRA) leads to synergistic attenuation of tumor growth in vitro and in xenograft models
Li et al., 2024 [39]	MF	45	scRNA-seq, TCR-seq, Bulk RNA-seq, WGS, Spatial transcriptomics, IF, IHC, CNV, cell–cell interactions, druggable target prediction	- For early and advanced-stage CTCL, gene expression is similar to central memory cells (SELL, CCR7, LEF1, and TCF7). - CTCL showed greatest enrichment in fibroblast subtype F2 and vascular endothelial subtype VE3. - CTCL enriched DCs had higher expression of LGAL59 and TNFSF12. - B cells are more abundant in CTCL skin samples than healthy skin, lesional and non-lesional AD and psoriatic skin.
Jiang et al., 2024 [27]	MF, SS	25	scRNA-seq, TCR-seq, custom gene panel, trajectory analysis, SMART-seq, Cell proliferation	- Panel of 19 genes accurately differentiated CTCL cells from non-malignant cells in blood and skin tissue. - Identified 3 phenostates of SS cells based on exhaustive markers and proliferative potential. - Trajectory analysis revealed SS as distinct from MF.
Jiang et al., 2024 [42]	MF, SS (Relapsed/ Refractory)	9	scRNA-seq, TCR-seq, custom gene panel, Monocyte phagocytosis assay, FC	- Role of dysfunctional monocytes in SS pathogenesis with decreased phagocytosis and chemokine response. - Synergistic effect of anti-CCR4 and interferon-α2a in improving phagocytosis and modulating macrophage activation.
Shi et al., 2024 [28]	MF, FMF	2	scRNA-seq	- Cluster of fibroblasts present in FMF but not MF. - Exclusive markers (CCL19, CRABP1, ADAMTS8, CCL26, CP, PLA2G2A, STEAP2, RBP5, PTX3 and ADH1B) were related to ECM structure, cell adhesion and cell chemotaxis.
Calugareanu et al., 2023 [30]	Advanced MF, eMF, SS, pcAECyTCL	5	scRNA-seq, cell–cell communication	- Phenotypic malignant cell-driven switch from early-stage CTCL (Th1 polarization) to later stages (Th2). - Higher proportion of KCs and lower number of fibroblasts in CTCL.
Gaydosik et al., 2023 [31]	Advanced MF	10	scRNA-seq, pathway analysis, cell–cell communication, IHC	- Wide interpatient heterogeneity in gene expression and proportion of cell types. - MF-specific TME compared to HC or BD, with alterations in immune functions, cell trafficking, angiogenesis, matrix interactions, and metabolism pathways.
Harro et al., 2023 [23]	MF, SS	7	scRNA-seq, TCR-seq, scATAC-seq, qPCR, WES	- CTCL cells likely originate from mutated hematopoietic progenitor cells after thymic egress.
Gao et al., 2023 [44]	Advanced MF	1	scRNA-seq, TCR-seq, WGS, WES, FC, ex vivo cell assay	- PD-1 may be a tumor suppressor of malignant T cells with TCR activation - PD-1 blockade may induce proliferation of malignant CD4+ T-cells with functional PD-1 expression and an exhausted status
Ren et al., 2023 [26]	MF, SS	11	scRNA-seq, TCR-seq, PHATE, CNV, SNV, WES, WGS	- Found circulating presumed precancerous clonal CD4+ T-cell population
Borcherding et al., 2023 [25]	SS	6	scRNA-seq, TCR-seq, Bulk RNA-seq, IHC, FC	- AIRE was the most upregulated gene across CTCLs. - Intra-patient analysis showed increased FOXP3 expression after HDACi and photopheresis treatment.
Du et al., 2022 [43]	SS, LCT	5	scRNA-seq, trajectory analysis, CNV, cell–cell communication	- CTCL had a higher enrichment of T/NK and myeloid cells. - Malignant T cell sub-population (CXCR3+, GNLY+, CREM+, and MKI67+) with high proliferation, stemness, and copy number variation (CNV) levels - CCL13+ monocytes/macrophages and LAMP3+ cDC cells were enriched and mediated immunosuppression via inhibitory interactions (CD47-SIRPA, MIF-CD74, and CCR1-CCL18) with malignant T cells.
Xue et al., 2022 [32]	SS	1	scRNA-seq, scTCR-seq, scATAC-seq, validation with qRT-PCR, FC, IHC, in vitro cell culture	- Identified specific marker genes (TOX, DNM3, KLHL42, PGM2L1, and SESN3) shared in blood- and skin-derived Sézary cells.
Su et al., 2022 [53]	SS	6	scRNA-seq, Bulk RNA-seq, TCR-seq, trajectory analysis, SNV, CNV, Flow cytometry	- Pembrolizumab responders had higher expression of KIF5B, HSB17B11, lower expression of KIR3DL2, FYB1, TBC1D10C, and ARPC1B, TCF7.

Abbreviations: # (number), AD (Atopic dermatitis), CIE (Chronic idiopathic erythroderma), CNV (Copy number variation), CODEX (Co-detection by indexing; multiplexed tissue imaging), eCTCL (Erythrodermic cutaneous T-cell lymphoma), esMF (Early-stage mycosis fungoides), GSEA (Gene set enrichment analysis), IHC (Immunohistochemistry), FC (Flow cytometry), FMF (Folliculotropic mycosis fungoides), HC (Healthy control), LCT (Large-cell transformation), LPP (Large-plaque parapsoriasis), MF (Mycosis fungoides), pcAECyTCL (Primary cutaneous aggressive epidermotropic cytotoxic T-cell lymphoma), PHATE (potential of heat diffusion for affinity-based trajectory embedding analysis), PP (Parapsoriasis), qRT-PCR (quantitative real-time reverse transcriptase polymerase chain reaction), scRNA-seq (Single-cell RNA sequencing), SNV (single nucleotide variants), SPP (Small-plaque parapsoriasis), TCR-seq (T-cell receptor sequencing), WES (whole-exome sequencing), WGS (whole-genome sequencing).

## References

[1] Willemze R., Cerroni L., Kempf W., Berti E., Facchetti F., Swerdlow S.H., Jaffe E.S. (2019). The 2018 update of the WHO-EORTC classification for primary cutaneous lymphomas. Blood.

[2] Campo E., Jaffe E.S., Cook J.R., Quintanilla-Martinez L., Swerdlow S.H., Anderson K.C., Brousset P., Cerroni L., de Leval L., Dirnhofer S. (2022). The International Consensus Classification of Mature Lymphoid Neoplasms: A report from the Clinical Advisory Committee. Blood.

[3] Alaggio R., Amador C., Anagnostopoulos I., Attygalle A.D., Araujo I.B.O., Berti E., Bhagat G., Borges A.M., Boyer D., Calaminici M. (2022). The 5th edition of the World Health Organization Classification of Haematolymphoid Tumours: Lymphoid Neoplasms. Leukemia.

[4] Hristov A.C., Tejasvi T., Wilcox R.A. (2025). Mycosis Fungoides, Sézary Syndrome, and Cutaneous B-Cell Lymphomas: 2025 Update on Diagnosis, Risk-Stratification, and Management. Am. J. Hematol..

[5] Cai Z.R., Chen M.L., Weinstock M.A., Kim Y.H., Novoa R.A., Linos E. (2022). Incidence Trends of Primary Cutaneous T-Cell Lymphoma in the US From 2000 to 2018: A SEER Population Data Analysis. JAMA Oncol..

[6] Tensen C.P., Quint K.D., Vermeer M.H. (2022). Genetic and epigenetic insights into cutaneous T-cell lymphoma. Blood.

[7] Park J., Daniels J., Wartewig T., Ringbloom K.G., Martinez-Escala M.E., Choi S., Thomas J.J., Doukas P.G., Yang J., Snowden C. (2021). Integrated genomic analyses of cutaneous T-cell lymphomas reveal the molecular bases for disease heterogeneity. Blood.

[8] Bakr F.S., Whittaker S.J. (2022). Advances in the understanding and treatment of Cutaneous T-cell Lymphoma. Front. Oncol..

[9] Hristov A.C., Tejasvi T., Wilcox R.A. (2021). Cutaneous T-cell lymphomas: 2021 update on diagnosis, risk-stratification, and management. Am. J. Hematol..

[10] Tang F., Barbacioru C., Wang Y., Nordman E., Lee C., Xu N., Wang X., Bodeau J., Tuch B.B., Siddiqui A. (2009). mRNA-Seq whole-transcriptome analysis of a single cell. Nat. Methods.

[11] Tirosh I., Izar B., Prakadan S.M., Wadsworth M.H., Treacy D., Trombetta J.J., Rotem A., Rodman C., Lian C., Murphy G. (2016). Dissecting the multicellular ecosystem of metastatic melanoma by single-cell RNA-seq. Science.

[12] Steen C.B., Luca B.A., Esfahani M.S., Azizi A., Sworder B.J., Nabet B.Y., Kurtz D.M., Liu C.L., Khameneh F., Advani R.H. (2021). The landscape of tumor cell states and ecosystems in diffuse large B cell lymphoma. Cancer Cell.

[13] Andor N., Simonds E.F., Czerwinski D.K., Chen J., Grimes S.M., Wood-Bouwens C., Zheng G.X.Y., Kubit M.A., Greer S., Weiss W.A. (2019). Single-cell RNA-Seq of follicular lymphoma reveals malignant B-cell types and coexpression of T-cell immune checkpoints. Blood.

[14] Aoki T., Chong L.C., Takata K., Milne K., Hav M., Colombo A., Chavez E.A., Nissen M., Wang X., Miyata-Takata T. (2020). Single-Cell Transcriptome Analysis Reveals Disease-Defining T-cell Subsets in the Tumor Microenvironment of Classic Hodgkin Lymphoma. Cancer Discov..

[15] Gaydosik A.M., Tabib T., Geskin L.J., Bayan C.A., Conway J.F., Lafyatis R., Fuschiotti P. (2019). Single-Cell Lymphocyte Heterogeneity in Advanced Cutaneous T-cell Lymphoma Skin Tumors. Clin. Cancer Res..

[16] Mimitou E.P., Cheng A., Montalbano A., Hao S., Stoeckius M., Legut M., Roush T., Herrera A., Papalexi E., Ouyang Z. (2019). Multiplexed detection of proteins, transcriptomes, clonotypes and CRISPR perturbations in single cells. Nat. Methods.

[17] Rindler K., Jonak C., Alkon N., Thaler F.M., Kurz H., Shaw L.E., Stingl G., Weninger W., Halbritter F., Bauer W.M. (2021). Single-cell RNA sequencing reveals markers of disease progression in primary cutaneous T-cell lymphoma. Mol. Cancer.

[18] Herrera A., Cheng A., Mimitou E.P., Seffens A., George D., Bar-Natan M., Heguy A., Ruggles K.V., Scher J.U., Hymes K. (2021). Multimodal single-cell analysis of cutaneous T-cell lymphoma reveals distinct subclonal tissue-dependent signatures. Blood.

[19] Jonak C., Alkon N., Rindler K., Rojahn T.B., Shaw L.E., Porkert S., Weninger W., Trautinger F., Stingl G., Tschandl P. (2021). Single-cell RNA sequencing profiling in a patient with discordant primary cutaneous B-cell and T-cell lymphoma reveals micromilieu-driven immune skewing. Br. J. Dermatol..

[20] Li Z., Wang H., Dong R., Man J., Sun L., Qian X., Zhu X., Cao P., Yu Y., Le J. (2021). Single-Cell RNA-seq Reveals Characteristics of Malignant Cells and Immune Microenvironment in Subcutaneous Panniculitis-Like T-Cell Lymphoma. Front. Oncol..

[21] Borcherding N., Voigt A.P., Liu V., Link B.K., Zhang W., Jabbari A. (2019). Single-Cell Profiling of Cutaneous T-Cell Lymphoma Reveals Underlying Heterogeneity Associated with Disease Progression. Clin. Cancer Res..

[22] Buus T.B., Willerslev-Olsen A., Fredholm S., Blümel E., Nastasi C., Gluud M., Hu T., Lindahl L.M., Iversen L., Fogh H. (2018). Single-cell heterogeneity in Sézary syndrome. Blood Adv..

[23] Harro C.M., Sprenger K.B., Chaurio R.A., Powers J.J., Innamarato P., Anadon C.M., Zhang Y., Biswas S., Mandal G., Mine J.A. (2023). Sézary syndrome originates from heavily mutated hematopoietic progenitors. Blood Adv..

[24] Alkon N., Chennareddy S., Cohenour E.R., Ruggiero J.R., Stingl G., Bangert C., Rindler K., Bauer W.M., Weninger W., Griss J. (2025). Single-cell sequencing delineates T-cell clonality and pathogenesis of the parapsoriasis disease group. J. Allergy Clin. Immunol..

[25] Borcherding N., Severson K.J., Henderson N., Ortolan L.S., Rosenthal A.C., Bellizzi A.M., Liu V., Link B.K., Mangold A.R., Jabbari A. (2023). Single-cell analysis of Sézary syndrome reveals novel markers and shifting gene profiles associated with treatment. Blood Adv..

[26] Ren J., Qu R., Rahman N.T., Lewis J.M., King A.L.O., Liao X., Mirza F.N., Carlson K.R., Huang Y., Gigante S. (2023). Integrated transcriptome and trajectory analysis of cutaneous T-cell lymphoma identifies putative precancer populations. Blood Adv..

[27] Jiang T.T., Cao S., Kruglov O., Virmani A., Geskin L.J., Falo L.D., Akilov O.E. (2024). Deciphering Tumor Cell Evolution in Cutaneous T-Cell Lymphomas: Distinct Differentiation Trajectories in Mycosis Fungoides and Sézary Syndrome. J. Investig. Dermatol..

[28] Shi H.Z., Tian C.C., Kong Y.Q., Sun J.F., Chen H. (2024). Single-cell RNA sequencing reveals the underlying mechanism of folliculotropism in folliculotropic mycosis fungoides. Exp. Dermatol..

[29] Kwantwi L.B., Rosen S.T., Querfeld C. (2024). The Tumor Microenvironment as a Therapeutic Target in Cutaneous T Cell Lymphoma. Cancers.

[30] Calugareanu A., de Masson A., Battistella M., Michel L., Ram-Wolff C., Bouaziz J.D., Peltier S., Bensussan A., Bagot M., Dobos G. (2023). Exploring the Nonlymphocytic Cutaneous Microenvironment in Advanced Cutaneous T-Cell Lymphomas using Single-Cell RNA Sequencing. J. Investig. Dermatol..

[31] Gaydosik A.M., Stonesifer C.J., Tabib T., Lafyatis R., Geskin L.J., Fuschiotti P. (2023). The mycosis fungoides cutaneous microenvironment shapes dysfunctional cell trafficking, antitumor immunity, matrix interactions, and angiogenesis. JCI Insight.

[32] Xue X., Wang Z., Mi Z., Liu T., Wang C., Shi P., Sun L., Yang Y., Li W., Wang Z. (2022). Single-cell analyses reveal novel molecular signatures and pathogenesis in cutaneous T cell lymphoma. Cell Death Dis..

[33] Hirahara K., Poholek A., Vahedi G., Laurence A., Kanno Y., Milner J.D., O’Shea J.J. (2013). Mechanisms underlying helper T-cell plasticity: Implications for immune-mediated disease. J. Allergy Clin. Immunol..

[34] Chennareddy S., Rindler K., Ruggiero J.R., Alkon N., Cohenour E.R., Tran S., Weninger W., Griss J., Jonak C., Brunner P.M. (2025). Single-cell RNA sequencing comparison of CD4+, CD8+ and T-cell receptor γδ+ cutaneous T-cell lymphomas reveals subset-specific molecular phenotypes. Br. J. Dermatol..

[35] Chennareddy S., Rindler K., Meledathu S., Naidu M.P., Alkon N., Ruggiero J.R., Szmolyan L., Weninger W., Bauer W.M., Griss J. (2025). Single-cell RNA sequencing of chronic idiopathic erythroderma defines disease-specific markers. J. Allergy Clin. Immunol..

[36] Jung J.M., Won C.H., Chang S.E., Lee M.W., Lee W.J. (2025). Spatially Resolved Single-Cell Transcriptome Analysis of Mycosis Fungoides Reveals Distinct Biomarkers GNLY and FYB1 Compared With Psoriasis and Chronic Spongiotic Dermatitis. Mod. Pathol..

[37] Luo C.H., Hu L.H., Liu J.Y., Xia L., Zhou L., Sun R.H., Lin C.C., Qiu X., Jiang B., Yang M.Y. (2024). CDK9 recruits HUWE1 to degrade RARα and offers therapeutic opportunities for cutaneous T-cell lymphoma. Nat. Commun..

[38] Srinivas N., Peiffer L., Horny K., Lei K.C., Buus T.B., Kubat L., Luo M., Yin M., Spassova I., Sucker A. (2024). Single-cell RNA and T-cell receptor sequencing unveil mycosis fungoides heterogeneity and a possible gene signature. Front. Oncol..

[39] Li R., Strobl J., Poyner E.F.M., Balbaa A., Torabi F., Mazin P.V., Chipampe N.J., Stephenson E., Ramírez-Suástegi C., Shanmugiah V.B.M. (2024). Cutaneous T cell lymphoma atlas reveals malignant T(H)2 cells supported by a B cell-rich tumor microenvironment. Nat. Immunol..

[40] Cabrera-Perez J.S., Carey V.J., Odejide O.O., Singh S., Kupper T.S., Pillai S.S., Weiss S.T., Akenroye A. (2025). Integrative epidemiology and immunotranscriptomics uncover a risk and potential mechanism for cutaneous lymphoma unmasking or progression with dupilumab therapy. J. Allergy Clin. Immunol..

[41] Zhao Y., Li Y., Wang P., Zhu M., Wang J., Xie B., Tang C., Ma Y., Wang S., Jin S. (2024). The cancer-associated fibroblasts interact with malignant T cells in mycosis fungoides and promote the disease progression. Front. Immunol..

[42] Jiang T.T., Kruglov O., Akilov O.E. (2024). Unleashed monocytic engagement in Sézary syndrome during the combination of anti-CCR4 antibody with type I interferon. Blood Adv..

[43] Du Y., Cai Y., Lv Y., Zhang L., Yang H., Liu Q., Hong M., Teng Y., Tang W., Ma R. (2022). Single-cell RNA sequencing unveils the communications between malignant T and myeloid cells contributing to tumor growth and immunosuppression in cutaneous T-cell lymphoma. Cancer Lett..

[44] Gao Y., Hu S., Li R., Jin S., Liu F., Liu X., Li Y., Yan Y., Liu W., Gong J. (2023). Hyperprogression of cutaneous T cell lymphoma after anti-PD-1 treatment. JCI Insight.

[45] Roccuzzo G., Giordano S., Fava P., Pileri A., Guglielmo A., Tonella L., Sanlorenzo M., Ribero S., Fierro M.T., Quaglino P. (2021). Immune Check Point Inhibitors in Primary Cutaneous T-Cell Lymphomas: Biologic Rationale, Clinical Results and Future Perspectives. Front. Oncol..

[46] Lesokhin A.M., Ansell S.M., Armand P., Scott E.C., Halwani A., Gutierrez M., Millenson M.M., Cohen A.D., Schuster S.J., Lebovic D. (2016). Nivolumab in Patients With Relapsed or Refractory Hematologic Malignancy: Preliminary Results of a Phase Ib Study. J. Clin. Oncol..

[47] Narducci M.G., Tosi A., Frezzolini A., Scala E., Passarelli F., Bonmassar L., Monopoli A., Accetturi M.P., Cantonetti M., Antonini Cappellini G.C. (2020). Reduction of T Lymphoma Cells and Immunological Invigoration in a Patient Concurrently Affected by Melanoma and Sezary Syndrome Treated With Nivolumab. Front. Immunol..

[48] Khodadoust M.S., Rook A.H., Porcu P., Foss F., Moskowitz A.J., Shustov A., Shanbhag S., Sokol L., Fling S.P., Ramchurren N. (2020). Pembrolizumab in Relapsed and Refractory Mycosis Fungoides and Sézary Syndrome: A Multicenter Phase II Study. J. Clin. Oncol..

[49] Marchi E., Ma H., Montanari F., Sawas A., Lue J.K., Deng C., Whitfield K.T., Klein S., Scotto L., Jain S.S. (2020). The Integration of PD1 blockade with epigenetic therapy is highly active and safe in heavily treated patients with T-cell lymphoma (PTCL) and cutaneous T-cell lymphoma (CTCL). J. Clin. Oncol..

[50] Querfeld C., Palmer J., Han Z., Wu X., Yuan Y.C., Chen M.H., Su C., Tsai N.C., Smith D.L., Hammond S.N. (2025). Phase 1 trial of durvalumab (anti-PD-L1) combined with lenalidomide in relapsed/refractory cutaneous T-cell lymphoma. Blood Adv..

[51] Querfeld C., Thompson J.A., Taylor M.H., DeSimone J.A., Zain J.M., Shustov A.R., Johns C., McCann S., Lin G.H.Y., Petrova P.S. (2021). Intralesional TTI-621, a novel biologic targeting the innate immune checkpoint CD47, in patients with relapsed or refractory mycosis fungoides or Sézary syndrome; syndrome: A multicentre, phase 1 study. Lancet Haematol..

[52] Costanza M., Giordano C., von Brünneck A.-C., Zhao J., Makky A., Vinh K., Montes-Mojarro I.A., Reisinger F., Forchhammer S., Witalisz-Siepracka A. (2025). Preclinical in vitro and in vivo evidence for targeting CD74 as an effective treatment strategy for cutaneous T-cell lymphomas. Br. J. Dermatol..

[53] Su T., Duran G.E., Kwang A.C., Ramchurren N., Fling S.P., Kim Y.H., Khodadoust M.S. (2022). Single-cell RNA-sequencing reveals predictive features of response to pembrolizumab in Sézary syndrome. OncoImmunology.

[54] Qin T., Billi A., Runge J., Wasikowski R., Li Q., Wang Y., Sartor M., Harms P.W., Gudjonsson J., Hristov A. (2023). 311 Single-cell transcriptomics reveals distinct molecular programs in folliculotropic mycosis fungoides. J. Investig. Dermatol..

[55] Wang C., Wolfe A., Geng X., Wilcox R.A. (2024). Single Cell Atlas of Cutaneous T Cell Lymphomas Reveals XPO1 Dependency. Blood.

[56] Childs B.A., Elghonaimy E., Aguilera T., Goff H.W. (2025). 0767 Characterizing malignant T cells in CTCL to inform personalized therapy: A scRNAseq study. J. Investig. Dermatol..

[57] Childs B., Elghonaimy E., Aguilera T., Goff H. (2025). 64086 Dissecting Malignant T-Cell Behavior in CTCL: A Single-Cell RNA Sequencing Comparison with Benign T Cells in the Immune Microenvironment. J. Am. Acad. Dermatol..

[58] Childs B., Elghonaimy E., Ramakrishnan Geethakumari P., Aguilera T., Goff H.W. (2024). Single-Cell Insights into Cellular Heterogeneity and Immune Dynamics in Cutaneous T-Cell Lymphoma. Blood.

[59] Johnson C., Solhjoo S., Li W., Ali I., Nash K., Hicks S., Timp W. (2024). 099 Single-cell RNA sequencing reveals differential gene expression of cancer-associated fibroblast markers in mycosis fungoides by stage and race. J. Investig. Dermatol..

[60] Rassek K., Iżykowska K. (2020). Single-Cell Heterogeneity of Cutaneous T-Cell Lymphomas Revealed Using RNA-Seq Technologies. Cancers.

[61] Haque A., Engel J., Teichmann S.A., Lönnberg T. (2017). A practical guide to single-cell RNA-sequencing for biomedical research and clinical applications. Genome Med..

[62] Luo Y., De Gruijl F.R., Vermeer M.H., Tensen C.P. (2024). “Next top” mouse models advancing CTCL research. Front. Cell Dev. Biol..

[63] Marx V. (2021). Method of the Year: Spatially resolved transcriptomics. Nat. Methods.

[64] Deconinck L., Cannoodt R., Saelens W., Deplancke B., Saeys Y. (2021). Recent advances in trajectory inference from single-cell omics data. Curr. Opin. Syst. Biol..

[65] Lähnemann D., Köster J., Szczurek E., McCarthy D.J., Hicks S.C., Robinson M.D., Vallejos C.A., Campbell K.R., Beerenwinkel N., Mahfouz A. (2020). Eleven grand challenges in single-cell data science. Genome Biol..

[66] Liu X., Jin S., Hu S., Li R., Pan H., Liu Y., Lai P., Xu D., Sun J., Liu Z. (2022). Single-cell transcriptomics links malignant T cells to the tumor immune landscape in cutaneous T cell lymphoma. Nat. Commun..

